# A Clinical Review and Experience of Splenic Trauma in North India: A Retrospective Observational Study

**DOI:** 10.7759/cureus.55384

**Published:** 2024-03-02

**Authors:** Sunil Kumar, Vivek Katiyar, Sumit Sharma, Vipul K Srivastava, Satyanam K Bhartiya, Shashi P Mishra

**Affiliations:** 1 Division of Trauma Surgery, Department of General Surgery, Institute of Medical Sciences, Banaras Hindu University, Varanasi, IND

**Keywords:** mortality, interventions, splenectomy, abdominal injury, trauma

## Abstract

Introduction: The spleen is one of the frequently injured solid organs in abdominal blunt trauma. The standard of care is nonoperative nowadays depending on the hemodynamic stability (World Society of Emergency Surgery (WSES) grade I-III) of the patient due to advancements in treating modalities. Operative interventions are required in hemodynamically unstable patients or failure of nonoperative management. The study was planned to find the clinical spectrum of abdominal blunt trauma, specifically those having splenic trauma, and their subsequent management in an institution.

Methods: This is a retrospective observational study. All included patients with blunt abdominal injuries were treated in a level 1 trauma center between July 2021 and December 2022. Data regarding demographic profile, blood transfusion, pre- and postoperative findings, and management including the period of hospital stay, morbidity, and mortality were collected and analyzed.

Results: One hundred sixty-four patients were analyzed, of which 142 were males and 22 were females. The commonest mechanism of injury was motor vehicle collision, followed by falls. Grade III splenic injury was the most common injury, while the predominantly associated injury was rib fracture. The patients were managed preferably through nonoperative management, followed by angioembolization and operative management. The commonest postoperative complication was pneumonia.

Conclusions: Nonoperative management of splenic trauma has evolved as the standard of care replacing operative management in order to sustain its immune function, thereby preventing overwhelming post-splenectomy infection.

## Introduction

Nowadays, trauma is one of the leading causes of morbidity and mortality not only in developing countries but also worldwide [1,2]. Motor vehicle collision is the major cause of trauma in adults. From the worldwide analysis, mortality due to trauma is higher than any other individual disease mortality. Annually, more than 20 million people suffer from trauma worldwide, and injuries and violence take the lives of some 12,000 people around the world each day according to the World Health Organization report in November 2022.

Blunt trauma is one of the commonest modes of abdominal trauma [3]. Due to high splenic vascularity, it is vulnerable to high morbidity and mortality. The mortality rate from splenic trauma was reported as 13% or higher in many different studies, and this mortality rate is due to associated injuries [4].

The most commonly affected population due to trauma is the middle age population in both the developed and developing world [5]. Further management, which is either operative or nonoperative, depends on the type of trauma, grading of splenic injury, associated injury, severity, and mainly hemodynamic stability of patients. For the diagnosis of splenic trauma, contrast-enhanced computed tomography (CECT) imaging is the noninvasive diagnostic tool for abdominal blunt trauma [6].

Currently, the standard of choice is nonoperative management for splenic trauma in hemodynamically stable or rapid responder patients without other associated injuries that require surgery [7]. In higher-grade splenic injury, nonoperative management should only be attempted in a center with an operating room available 24/7, a blood bank, an intensive care unit (ICU), and an interventional radiology department [7].

According to recently published guidelines, the hemodynamic status of splenic trauma patients is the main deciding factor for nonoperative management [7]. Splenectomy is indicated only in selected patients with unstable hemodynamic status. The nonoperative management is due to the advancement and newer techniques of embolization and better monitoring systems.

The aim and objective of this study were to evaluate splenic trauma and various clinical presentations, along with modalities of its management approaches, and analyze outcomes and complications of splenic trauma.

## Materials and methods

This is a retrospective observational study of a single center that includes all patients admitted in the trauma surgery division of a level 1 trauma center in North India serving more than 200 million populations from July 2021 to December 2022. Hemodynamically unstable patients and nonresponders to fluid resuscitation with a positive extended focused assessment sonography in trauma (e-FAST) were directly shifted to the operating room and underwent emergency surgical procedures.

Inclusion criteria

Patients admitted to the trauma center with splenic injury (isolated or polytrauma) of both genders and ages more than 12 years were included. Patients who have a history of bleeding diathesis/gross splenomegaly/portal hypertension and are critically ill, i.e., hemodynamically unstable patients or patients with severe head injuries, were excluded from the study.

A retrospective trauma registry was reviewed for data analysis. All patients were managed according to Advanced Trauma Life Support (ATLS) guidelines. All polytrauma patients presented to the emergency department with suspicion of an intra-abdominal injury on initial assessment and were subjected to FAST after initiating resuscitation. Patients who attended the triage area were admitted after resuscitation and after confirming the diagnosis and shifted to the intervention room/ICU/ward according to the clinical status of the patients. Data of relevant findings including mechanism of injury, age, gender, duration of presentation, clinical parameters, laboratory parameters, imaging findings, intervention, outcome, and hospital stay were collected. All patients with suspected abdominal injury were assessed for abdominal visceral injury including splenic injury with clinical, biochemical, and imaging modalities at the time of presentation and thereafter depending on the patient's condition. The collected data was analyzed according to the Injury Severity Score (ISS) to reduce bias. All hemodynamically stable patients underwent a CECT scan to diagnose the injury and were graded using the American Association for Surgery of Trauma (AAST) grading system. Splenic injuries more than grade I were included in the study. All hemodynamically stable patients without other hollow organ injuries were chosen for nonoperative management. Patients with hemoperitoneum as per e-FAST and were hemodynamically unstable after resuscitation or those with clear signs of hollow viscus injury were taken up for exploration without subjecting them to a CECT scan.

The outcome was noted in the form of nonoperative management, intervention required, complications, morbidity, and mortality. Imaging was done at the time of presentation and post-injury on day 7. All emergency splenectomy patients were given a polyvalent vaccine on postoperative (PO) day 14 of surgery. Postoperative patients with no complications were discharged on the eighth postoperative (PO) day after imaging. Those who developed complications were discharged after complete recovery. Biochemical parameters include blood group, with hemoglobin, liver function, serum amylase serum lipase, and renal function test at the presentation and day 5 post-injury or depending on clinical parameters. All patients were discharged after satisfactory clinical status with biochemical parameters on post-trauma days 5-14 depending on patient status and were followed up. All relevant data were collected, analyzed, and interpreted.

Statistical analysis

Descriptive data analysis was used for interpretation, and results were expressed as mean, median, or percentage (%). Each variable outcome was examined individually in a univariate analysis considering nonoperative management, operative management, morbidity, and mortality as dependent variables.

## Results

Observations

All patients who were admitted to the trauma center fulfilling the inclusion criteria were included in this study. A total of 164 patients with splenic trauma, in the age group between 12 and 74 years, with an AAST grade of more than one, were included in this study. One hundred forty-two (86.58%) patients were male, and 22 (13.41%) were female. The adult population was more prone to trauma. The mean age was 37 years. The commonest mechanism of injury was vehicle collision (109 (66.46%)), followed by fall from height (28 (17.07%)), physical assault (13 (7.92%)), firearm injury (7 (4.26%)), animal injury (5 (3.04%)), blast injury (1 (0.6%)), and electric injury (1 (0.6%)) (Table 1). The time of presentation was between 30 minutes and five days. The mean time of presentation was nine hours (Table 1).

**Table 1:** Descriptive parameters of patients

Characteristics	Number	Percentage (%)
Mode of injury		
Road traffic accidents	109	66.46
Fall	28	17.07
Physical assault	13	7.92
Firearm	7	4.26
Animal	5	3.04
Electric	1	0.6
Blast	1	0.6
Age distribution		
12-30	75	45.73
31-50	64	39.02
>50	25	15.29
Grading of injury		
Grade II	48	29.26
Grade III	66	40.24
Grade IV	39	24.07
Grade V	11	6.07
Injury Severity Score		
Mild (1-8)	11	6.07
Moderate (9-15)	62	37.80
Severe (16-24)	54	32.92
Profound (>25)	37	22.56
Surgical interventions		
Nonoperative	137	83.53
Angioembolization	8	4.87
Splenectomy	9	5.48
Miscellaneous		
Polytrauma	121	73.78
Chest trauma	109	66.46
Mortality	14	8.53
Time of presentation (mean)	9 hours	
Male:female	6:1	
Hospital stay (mean)	8.4 days	

One hundred twenty-one (73.78%) patients were polytrauma patients with chest trauma, liver injury, bowel injury, head injury, bone injury, and pelvic injury, while isolated splenic injury was seen in 43 (26.21%) patients. Chest injuries were present in 109 (66.46%) cases (Table 1), predominantly left-side chest injuries. There are four (2.43%) cases of splenic injury associated with diaphragmatic injury. Abdominal pain, abdominal distension, and/or hypotension were the main symptoms of presentation.

The grading of injury was done by CT scan findings at the time of presentation in all hemodynamically stable patients. Grade II injuries were seen in 48 (29.26%) patients, grade III injuries in 66 (40.24%) patients, and grade IV splenic injuries in 39 (24.07%) patients (Table 1). Eleven (6.70%) patients have grade V splenic injuries. Eight (4.87%) patients underwent angioembolization, and nine (5.48%) patients underwent splenectomy (Table 1). A total of 27 (16.46%) patients require operative management either due to splenic injury or other associated organ injuries including bowel perforation, diaphragmatic injury, and bladder injury. Most of the high-grade injuries underwent intervention. One hundred twenty (73.17%) patients require a blood transfusion due to hypotension and/or decreased hemoglobin. Overall mortality was 8.53% (14 patients) (Table 1) with or without polytrauma with ISS of more than 25. In splenectomy patients, the mortality was 22.2% (two patients), mainly due to septic complications, while in nonoperative cases, the mortality was 8.75% (12 patients), mainly due to failure of nonoperative management and being in the age group of more than 55 years with polytrauma. Complications included fever, abdominal distension, pain, anemia, pleural effusion, pneumonia, and breathlessness in 58 (35.36%) patients (mild to severe). According to the Injury Severity Score, 54 (32.92%) patients have high-grade injuries, 37 (22.56%) have profound injuries, and 62 (37.80%) have moderate-grade injuries (Table 1). The rest of the patients have mild injuries according to the Injury Severity Score. The mean hospital stay was 8.4 days (Table 1). Patients were advised to follow up regularly post-discharge on days 7, 15, 30, and 90.

## Discussion

The spleen is one of the commonly injured organs in abdominal blunt trauma [8]. Of polytrauma patients, 16%-23.8% have splenic injury with a mortality rate of 9.3% largely due to other associated injuries or high-grade and delayed treatments [3]. Nowadays, nonoperative management is used in most cases due to advancements in monitoring and availability of angioembolization [9]. The benefits of these modalities include reduction of economic burden, blood component transfusion, nontherapeutic exploration rate, intra-abdominal complication, morbidity, and mortality [9,10]. Furthermore, preservation of the spleen prevents patients from overwhelming post-splenectomy infection, which is a fatal condition in splenectomy patients [11].

In the present study, male predominance was seen. The most effective population (18-50 years) was the adult population. Motor vehicle collision is the most common mechanism of injury. The time of presentation varies from hour to days. Musetti et al. (2022) studied 193 patients with splenic trauma, of which 144 were males (75%) and the mean age was 48 years [12]. The commonest mode of injuries was motor vehicle collision, which included motor vehicle crashes (45%), motorcycle crashes (23%), falls (23%), pedestrians (5%), and crashes (4%). In another study by Grootenhaar et al. (2021) on the treatment and outcomes of splenic injuries in the Netherlands, the mean age of the studied population was 12 years, and the study population was predominantly males (63.1%) [13]. Furthermore, the study by Gad et al. (2018) comparing the surgical and nonsurgical treatment of splenic trauma found that the commonest mode of injury was motor vehicle collision (55%), followed by fall (35%) and animal injury (10%) in nonoperative treated patients [14]. In operative patients, the commonest mode of injury was motor vehicle collision (60%), fall (30%), animal injury (5%), and heavy object falling (5%). In another study by Osifo et al. (2007), the commonest mode of injury was motor vehicle collision (50%), and the second most common mechanism was fall from height [15]. However, in the study by Kristoffersen and Mooney (2007), fall from height was the main cause of injury [16].

Rode et al. (2021) studied 52 patients, of which 36 were males and 16 were females [4]. In their study, the most common mechanism was blunt injury in 21 cases; two cases were penetrating injuries with motor vehicle accidents (34%). The mean time of presentation was 25.2 ± 26.5 hours.

In our study, the maximum number of patients with splenic trauma was associated with other injuries, in which chest injury was the most common injury. Kasula et al. (2016) reported that the commonest associated injury was chest injury with rib fractures in 139 patients (90.26%) and hemothorax/pneumothorax in 112 patients (72.73%) [17]. Multiple injuries were also noted in the same patients. In the study by Rode et al. (2021), the most common associated injury was rib fracture (13%), followed by ileal perforation (8.5%) and head injury (8.5%), but isolated splenic injury was seen in almost half of the patients (53%). A few patients who needed urgent management were polytrauma patients with multiple organ injuries.

In this study, abdominal pain, abdominal distension, and/or hypotension, along with a history of trauma, were the main symptoms of presentation. Grading of injury was done using CT scan imaging in all hemodynamically stable patients at the time of presentation and intraoperatively in hemodynamically unstable patients. The majority of patients have grade III injuries (66 (40.24%)), followed by grade II injuries (48 (29.26%)), and grade IV splenic injuries (39 (24.07%)). Grade V splenic injuries were seen in 11 (6.70%) patients. Eight (4.87%) patients underwent angioembolization, and nine (5.48%) underwent splenectomy. Twenty-seven (16.46%) patients require operative management either due to splenic injury or other associated organ injuries, including bowel perforation, diaphragmatic injury, and bladder injury. Most of the patients with high-grade and severe injuries underwent intervention.

Kasula et al. reported that abdominal tenderness (85.06%), guarding, rigidity (78.57%), and distension (42.2%) were the commonest signs [17]. In their study, the majority of patients had grade III injuries (89 (58.94%)), while two (1.29%) patients had grade V injuries. Four (2.69%), 44 (29.13%), and 10 (6.49%) patients had grade I, II, and IV injuries, respectively, of which five (3.04%) underwent angioembolization and seven (4.26%) required splenectomy. A total of 12 (7.31%) patients required operative management either due to splenic injury or other associated organ injuries.

In the study by Musetti et al., the majority of patients had grade III injury (67 (35%)), and 19%, 24%, 18%, and 4% of patients had grade I, II, IV, and V, respectively. One hundred forty (72%) patients were managed conservatively in the nonoperative group, and 53 patients underwent emergency splenectomy in the operative group. The operative group has more patients with ISS > 15.

Renzulli et al. introduced splenic artery embolization in their study, and there was a significant decrease in surgery rates from 33.3% to 11.9% (P < 0.001), but there was an insignificant difference in the success rate of nonoperative management [18].

The study published by Alamri et al. (2017) reported that the majority of patients (173 (72.7%)) were managed nonoperatively, whereas 37 (15.5%) patients underwent splenectomy [19]. Twenty-eight (11.8%) patients had splenic artery embolization. In another study by Bagaria et al., 95 patients were managed nonoperatively depending on their hemodynamic status [20]. Angioembolization was done in 25 patients, 17 prophylactics and eight therapeutic, of which three were for contrast extravasation and five were for pseudoaneurysms in the splenic artery or its branches. Thirty-four patients were selected for operative management due to hemodynamic instability, of which three were nonresponders and 31 were transient responders. Splenorrhaphy was performed in one patient with a grade II injury, and splenectomy was performed in 33 patients.

In the present study, mortality was present in 14 (8.53%) patients, mainly high-grade injury, with or without polytrauma, with ISS of more than 25.

In their study, Jesani et al. (2019) reported that hospital mortality was 10.3%, with no significant difference in nonoperative and operative groups [21]. Cadeddu et al. studied 266 patients, and 21 died during the study period (11 in the operative management (OM) group and 10 in the nonoperative management (NOM) group) [22]. A 9.3% mortality rate was observed in the operative group, whereas the nonoperative group had a 6.8% mortality.

In the present study, complications were mainly fever, pleural effusion, pneumonia, and breathlessness in 58 (35.36%) patients (mild to severe). Based on the Injury Severity Score, most of the patients had moderate-grade injuries (62 (37.80%)), followed by high-grade injuries (54 (32.92%)), and profound injuries (37 (22.56%)). The rest of the patients have mild-grade injuries.

In the study by Musetti et al., the number of patients with ISS > 15 was higher in the operative group [12]. Fransvea et al. found that patients who underwent splenectomy had an average ISS of 30.88 ± 11.84, whereas the average ISS for nonoperative patients was 20.81 ± 12.12 [23].

In the present study, the mean hospital stay was 8.4 days in both operative and nonoperative groups and high-grade injury (ISS > 25).

The median length of hospital stay in the study by Alamri et al. was 6.8 days (0-190 days) [19]. Bagaria et al. reported that the nonoperative group has a median length of hospital stay of five days, whereas the surgical group has six days [20].

Chalya et al. and Weinberg et al. have reported their series without angioembolization and with contrasting data on operative versus nonoperative management [24,25].

A comparison of the various studies is shown in Table 2.

**Table 2:** Comparison of management and outcomes of the different studies NOM: nonoperative management, OM: operative management

Studies	Number of cases	NOM (%)	Angioembolization (%)	OM (%)	Mortality (%)
Musetti et al. [12]	193	72	-	28	5.18
Kasula et al. [17]	154	12.98	0	85.06	4.51
Alamri et al. [19]	238	72.7	11.8	15.5	8
Chalya et al. [24]	118	13.56	0	94.91	19.49
Weinberg et al. [25]	426	80.04	0	19.95	4
Bagaria et al. [20]	129	73.64	19.37	26.35	1.55
Present study	164	83.53	4.87	16.46	8.53

Limitations

The present study has limitations, including a small sample size, polytrauma, and a short study duration. The Trauma and Injury Severity Score (TRISS) is a more efficient scale than the ISS for predicting mortality in trauma patients. We will further increase the number of cases and use the TRISS to explain the prognosis and mortality of trauma patients.

## Conclusions

The management of splenic injury has evolved from operative to nonoperative as the standard of care over the past years to preserve its immune function, thereby preventing overwhelming post-splenectomy infection. Nonoperative management is primarily based on the hemodynamic status of patients irrespective of grading, exclusion of multisystem injury, and availability of diagnostic and operative facilities along with intervention and intensive care facilities. After the application of embolization and CT diagnosis, there has been a progressive increase in the success rate of nonoperative management. The use of diagnostic laparoscopy in hemodynamically stable splenic trauma reduces the unnecessary exploration that leads to increased morbidity.

Still, there is the question regarding the definitive patient selection criteria for arterial embolization and nonoperative management and their appropriate application. The formulation of a management protocol for severe high-grade injuries and failure of nonoperative management with a decrease in the complication of injury should be prioritized in further studies. To prove the benefit of minimally invasive splenic intervention, prospective trials with clear inclusion criteria are also needed.
